# Detection of quantitative trait loci affecting haematological traits in swine via genome scanning

**DOI:** 10.1186/1471-2156-11-56

**Published:** 2010-06-28

**Authors:** Yuan-Fang Gong, Xin Lu, Zhi-Peng Wang, Fang Hu, Yan-Ru Luo, Shao-Qian Cai, Chun-Mei Qi, Shan Li, Xiao-Yan Niu, Xiao-Tian Qiu, Jian Zeng, Qin Zhang

**Affiliations:** 1 Key Laboratory Animal Genetics and Breeding of the Ministry of Agriculture, College of Animal Science and Technology, China Agricultural University, Beijing 100193, China; 2 Department of Animal Science, Hebei Normal University of Science & Technology, Chang li, Hebei 066600, China; 3 National Institute for Communicable Disease Control and Prevention, Chinese Center for Disease Control and Prevention, P.O. Box 5, Changping, Beijing 102206, China

## Abstract

**Background:**

Haematological traits, which consist of mainly three components: leukocyte traits, erythrocyte traits and platelet traits, play extremely important role in animal immune function and disease resistance. But knowledge of the genetic background controlling variability of these traits is very limited, especially in swine.

**Results:**

In the present study, 18 haematological traits (7 leukocyte traits, 7 erythrocyte traits and 4 platelet traits) were measured in a pig resource population consisting of 368 purebred piglets of three breeds (Landrace, Large White and Songliao Black Pig), after inoculation with the swine fever vaccine when the pigs were 21 days old. A whole-genome scan of QTL for these traits was performed using 206 microsatellite markers covering all 18 autosomes and the X chromosome. Using variance component analysis based on a linear mixed model and the false discovery rate (FDR) test, 35 QTL with FDR < 0.10 were identified: 3 for the leukocyte traits, 28 for the erythrocyte traits, and 4 for the platelet traits. Of the 35 QTL, 25 were significant at *FDR *< 0.05 level, including 9 significant at *FDR *< 0.01 level.

**Conclusions:**

Very few QTL were previously identified for hematological traits of pigs and never in purebred populations. Most of the QTL detected here, in particular the QTL for the platelet traits, have not been reported before. Our results lay important foundation for identifying the causal genes underlying the hematological trait variations in pigs.

## Background

In pork-producing community, various infectious diseases caused by viral or bacterial pathogens badly restrict the efficiency of pork industries, and also seriously affect human health in the whole world. To most infectious diseases, the resistance of an individual is resulted from both innate immunity and acquired immunity. The capacity of innate immunity or acquired immunity is more or less controlled by genes [1-4]. The resistance to some diseases is due to a single gene [5,6], but for most diseases it is due to multiple genes [7], among which a few genes (QTL or major genes) have relative large effects and thus play important role in disease resistance. Therefore, detection of these genes or QTL is important for disease-resistant breeding.

Haematological traits, which consist of three components: leukocyte traits, erythrocyte traits, and platelet traits, are important indicators of immune capacity of animals [8-10]. The influence of genetics on baseline peripheral blood cell parameters has been firmly established. In human, the heritability estimates for red blood cell, white blood cell, and platelet numbers derived from a twin study [11] were 0.42, 0.62, and 0.57, respectively, indicating a large genetic component underlies these traits. Study in baboons [12] confirmed that genetic influences account for a significant portion of the variance in these blood parameters as well as in mean corpuscular volume (MCV) and mean platelet volume (MPV). In swine, significant differences in a panel of haematological traits between Meishan and Large White pigs were observed by Clapperton *et al*. [1], which may have implications in the resistance to infection by a broad range of pathogens and subsequent disease effects in these breeds. However, knowledge of the genetic background controlling the variability of haematological traits, is very limited, especially in swine.

With the continuously increasing of DNA molecular markers and deep development of pig genome research, a large number of QTL or major genes controlling quantitative traits in pigs have been found and located in the relevant regions of chromosomes [13-15]. However, compared to the production traits, QTL mapping for haematological traits has been carried out only in a few research groups. Edfors-Lilja *et al*. [16,17] first reported QTL on SSC1 and 8 for stress-mediated alterations in some leukocyte traits in swine, which were further confirmed later in another mapping study by the same group [18]. Reiner *et al*. [19] reported nine genome-wide significant and 29 putative QTL distributed on 15 chromosomes controlling baseline leukocyte traits and leukocyte traits at distinct stages of the disease model. QTL for erythroid traits were also reported by the same group [20]. 43 QTL controlling baseline erythroid traits and erythroid traits at distinct stages of the disease model were identified on 16 chromosomes, of which twelve were significant at the genome-wide level. In a very recent study [21], a total of 101 QTL, including 46 genome-wide significant QTL, regulating baseline erythroid traits at different age stages were found on all pig chromosomes except for SSC15 and SSC18. For platelet traits, there are no reports of QTL mapping studies in swine up to now.

The immune response to "stress" is important indicator of disease resistance capacity of animals. In the present study, we report the results of QTL mapping for the stress induced immune response of pigs which were challenged with swine fever vaccine. Unlike the previous studies mentioned above, where F2 animals derived from cross of two breeds was used as mapping population, we used purebred animals with half- and full-sib families as mapping population. 18 haematological traits, including seven leukocyte traits, seven erythrocyte traits, and four platelet traits, were measured after they were inoculated with the swine fever vaccine.

## Results

### Phenotypes

The means and standard errors of the phenotypic values of the 18 haematological traits in the mapping population are presented in Table 1. These results are generally consistent with previous results [19-21]. Significant differences (*P *< 0.05) between measurements before (on day 20) and after (on day 35) inoculation with the swine fever vaccine were observed for all traits except GR%, LY% and MO%. Differences between the three breeds are significant for most of the leukocyte traits and two of the four platelet traits, but not significant for all erythrocyte traits except MCHC. No significant differences between sexes were observed for all traits except RBC and MCHC.

**Table 1:** Means and standard errors of haematological traits in piglets

Trait*	Age^$^		Breed (35 days, LS means) ^$^			Sex (35 days, LS means) ^$^	
	20 days	35 days	Landrace	Large White	Songliao	Male	Female
Leukocyte traits							
WBC	12.11 ± 0.32^a^	18.35 ± 0.40^b^	16.44 ± 0.81^a^	17.60 ± 0.57^a^	21.39 ± 0.79^b^	18.19 ± 0.58^a^	18.75 ± 0.57^a^
GR	2.75 ± 0.14^a^	4.80 ± 0.27^b^	4.35 ± 0.54^a^	4.01 ± 0.37^b^	6.43 ± 0.51^c^	4.82 ± 0.37^a^	5.04 ± 0.37^a^
GR%	21.95 ± 0.82^a^	23.84 ± 0.93^a^	24.75 ± 1.73^a^	23.10 ± 1.19^a^	25.61 ± 1.65^a^	24.30 ± 1.18^a^	24.68 ± 1.20^a^
LY	7.41 ± 0.20^a^	10.79 ± 0.22^b^	9.89 ± 0.45^a^	10.42 ± 0.31^a^	11.78 ± 0.44^b^	10.55 ± 0.31^a^	10.84 ± 0.32^a^
LY%	62.24 ± 0.81^a^	60.91 ± 0.84^a^	61.21 ± 1.64^a^	61.39 ± 1.13^a^	58.73 ± 1.56^a^	60.95 ± 1.12^a^	59.93 ± 1.14^a^
MO	1.95 ± 0.07^a^	2.76 ± 0.08^b^	2.25 ± 0.16^a^	2.71 ± 0.11^b^	3.20 ± 0.16^c^	2.58 ± 0.11^a^	2.85 ± 0.11^a^
MO%	15.80 ± 0.37^a^	15.29 ± 0.33^a^	14.07 ± 0.64^a^	15.48 ± 0.44^b^	15.85 ± 0.61^b^	14.77 ± 0.43^a^	15.50 ± 0.44^a^
Erythrocyte traits							
RBC	5.57 ± 0.06^a^	6.18 ± 0.07^b^	6.34 ± 0.14^a^	6.03 ± 0.10^a^	6.26 ± 0.14^a^	6.37 ± 0.10^a^	6.05 ± 0.10^b^
HGB	113.49 ± 1.17^a^	118.26 ± 1.33^b^	116.32 ± 2.79^a^	118.63 ± 1.96^a^	119.83 ± 2.70^a^	117.97 ± 1.92^a^	118.54 ± 1.96^a^
HCT%	35.02 ± 0.37^a^	37.02 ± 0.41^b^	37.07 ± 0.84^a^	37.16 ± 0.59^a^	36.93 ± 0.82^a^	37.22 ± 0.58^a^	36.89 ± 0.59^a^
MCV	62.95 ± 0.28^a^	57.64 ± 0.27^b^	58.49 ± 0.45^a^	58.11 ± 0.31^a^	57.31 ± 0.43^a^	57.98 ± 0.31^a^	57.96 ± 0.31^a^
MCH	20.51 ± 0.11^a^	18.43 ± 0.08^b^	18.39 ± 0.17^a^	18.65 ± 0.12^a^	18.51 ± 0.16^a^	18.41 ± 0.12^a^	18.63 ± 0.12^a^
MCHC	325.02 ± 1.10^a^	320.45 ± 1.17^b^	313.99 ± 2.48^a^	319.35 ± 1.69^a^	327.07 ± 2.31^b^	317.71 ± 1.65^a^	322.56 ± 1.69^b^
RDW%	17.59 ± 0.17^a^	17.68 ± 0.18^b^	17.61 ± 0.26^a^	17.50 ± 0.18^a^	18.02 ± 0.25^a^	17.73 ± 0.18^a^	17.68 ± 0.18^a^
Platelet traits							
PLT	398.94 ± 9.80^a^	466.05 ± 10.90^b^	428.46 ± 21.81^a^	490.66 ± 15.20^b^	427.25 ± 21.15^a^	441.49 ± 14.90^a^	456.09 ± 15.33^a^
MPV	9.65 ± 0.07^a^	9.08 ± 0.08^b^	9.32 ± 0.14^a^	9.24 ± 0.09^a^	8.96 ± 0.13^a^	9.11 ± 0.09^a^	9.22 ± 0.10^a^
PDW	14.99 ± 0.07^a^	14.72 ± 0.07^b^	14.85 ± 0.13^a^	14.82 ± 0.09^a^	14.61 ± 0.13^a^	14.72 ± 0.09^a^	14.80 ± 0.09^a^
PCT	0.37 ± 0.01^a^	0.41 ± 0.01^b^	0.38 ± 0.02^a^	0.44 ± 0.01^b^	0.36 ± 0.02^a^	0.39 ± 0.01^a^	0.40 ± 0.01^a^

* WBC: white blood cell count, GR: neutrophilic granulocyte count, GR%: neutrophilic granulocyte, percentage, LY: lymphocyte count, LY%: lymphocyte count percentage, MO: monocyte count, MO%: monocyte count percentage, RBC: red blood cell count, HGB: hemoglobin, HCT: haematocrit, MCV: mean corpuscular volume, MCH: mean corpuscular hemoglobin, MCHC: mean corpuscular hemoglobin concentration, RDW: red blood cell volume distribution width, PLT: platelet count, MPV: mean platelet volume, PDW: platelet distribution width, PCT: plateletocrit

$ Means with different superscript letters are significantly different (***P *< 0.05**).

### QTL for the 18 haematological traits

**Figure 1:**
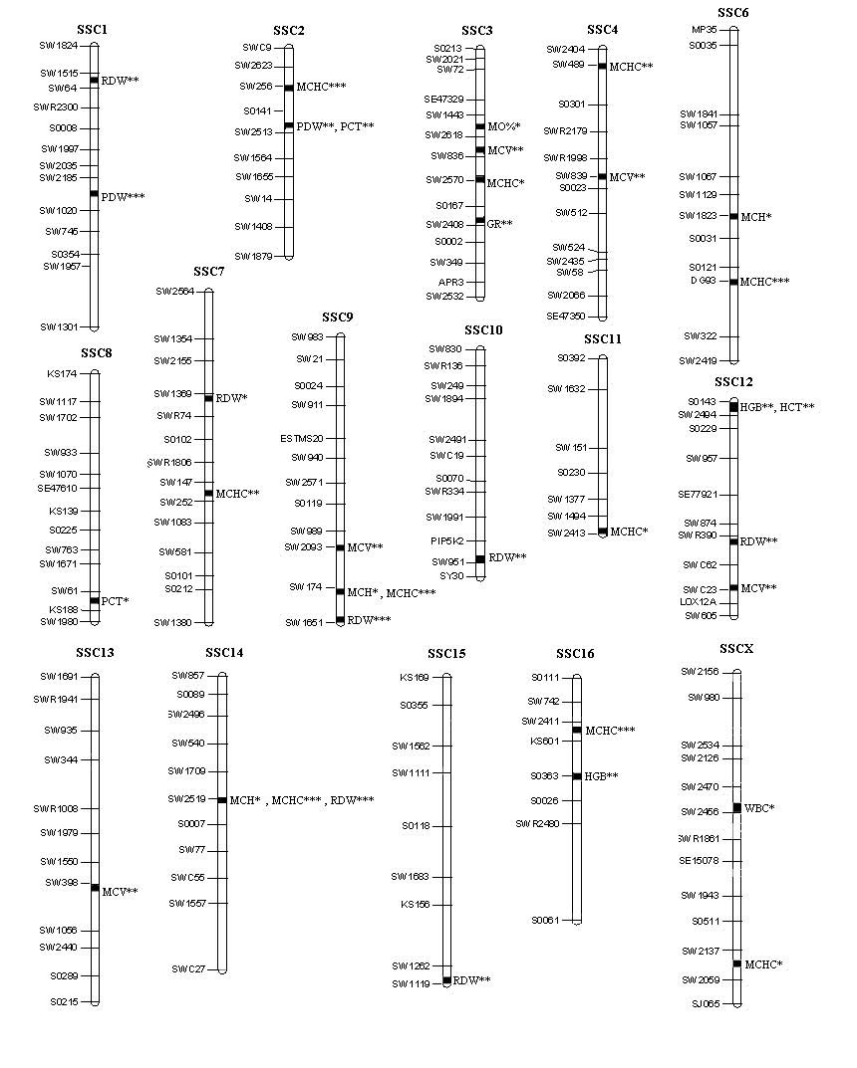
**Distribution of QTL in the porcine genome for leukocyte, erythrocyte and platelet traits after inoculation with the swine fever vaccine at 21 days old**. *significant with *FDR *< 0.10, **significant with *FDR *< 0.05, ***significant with *FDR *< 0.01

The QTL detected with *FDR *< 0.10 for the 18 haematological traits are presented in Table 2. For the leukocyte traits, only three QTL, including one with *FDR *< 0.05, were detected for GR, MO% and WBC, respectively. For the erythrocyte traits, 28 QTL, including 21 with *FDR *< 0.05, were detected: ten for MCHC, seven for RDW, five for MCV, three for MCH, two for HGB, and one for HCT. For the platelet traits, four QTL, including three with *FDR *< 0.05, were detected: two for PCT and two for PDW. The positions of all of the detected QTL are shown in Fig. 1.

**Table 2:** Results of QTL mapping for haematological traits

						Flanking markers	
						
Chr.	Trait	Position (cM)	LR value	P value ^a^	CI_95_^b ^(cM-cM)	Left	Right
**Leukocyte traits**							
3	GR	90	16.35	2.82E-04**	86.1-94.1	S0167	SW2408
	MO%	45	14.83	6.03E-04*	41.1-50.1	SW1443	SW2618
X	WBC	53	15.27	4.83E-04*	50.2-54.2	SW2470	SW2456
**Erythrocyte traits**							
1	RDW	20	20.63	3.31E-05**	17-22	SW1515	SW64
2	MCHC	20	21.30	2.37E-05***	15.6-24.6	SW256	S0141
3	MCV	57	17.59	1.51E-04**	53.1-61.1	SW2618	SW836
	MCHC	72	15.54	4.21E-04*	68.1-74.1	SW836	SW2570
4	MCV	62	20.63	3.32E-05**	59-65	SWR1998	SW938
	MCHC	9	18.62	9.05E-05**	7-13	SW489	S0301
6	MCH	91	14.08	8.75E-04*	90-94	SW1823	S0031
	MCHC	123	28.01	8.25E-07***	122-127	DG93	SW322
7	MCHC	95	17.43	1.64E-04**	91-98	SW147	SW252
	RDW	52	14.26	8.02E-04*	48-56	SW1369	SWR74
9	MCV	104	18.80	8.27E-05**	101-109	SW2093	SW174
	MCH	124	14.39	7.50E-04*	104-138	SW174	SW1651
	MCHC	124	33.20	6.18E-08***	114-130	SW174	SW1651
	RDW	138	22.57	1.25E-05***	135-138	SW174	SW1651
10	RDW	99	17.08	1.96E-04**	96-100	PIP5K2	SW951
11	MCHC	84	15.19	5.02E-04*	80.9-83.9	SW1494	SW2413
12	HGB	8	17.45	1.62E-04**	6.6-12.6	S0143	SW2494
	HCT	9	18.47	9.74E-05**	6.6-13.6	S0143	SW2494
	MCV	95	17.50	1.59E-04**	90.6-101.6	SWC62	SWC23
	RDW	74	16.55	2.55E-04**	68.6-78.6	SWR390	SWC62
13	MCV	81	16.31	2.87E-04**	76.2-87.2	SW398	SW1056
14	MCH	51	15.33	4.69E-04*	45.4-52.4	SW2519	S0007
	MCHC	51	26.19	2.06E-06***	49.4-53.4	SW2519	S0007
	RDW	51	23.95	6.27E-06***	47.4-52.4	SW2519	S0007
15	RDW	119	23.09	9.66E-06***	114-119	SW1262	SW1119
16	HGB	37	16.56	2.54E-04**	35-37	KS601	S0363
	MCHC	19	31.19	1.69E-07***	15-23	SW2411	KS601
X	MCHC	114	15.47	4.37E-04*	111.2-117.2	SW2137	SW2059
**platelet traits**							
1	PDW	75	22.24	1.48E-05***	70-83	SW2185	SW1020
2	PDW	38	19.12	7.06E-05**	30.6-45.6	S0141	SW2513
	PCT	38	17.89	1.30E-04**	30.6-47.6	S0141	SW2513
8	PCT	117	14.61	6.73E-04*	112.5-120.5	SW61	KS188

a: * *FDR *< 0.10; ** *FDR *< 0.05; *** *FDR *< 0.01

b: 95% confidence interval

## Discussion

So far, there are only limited reports on QTL mapping for haematological traits in pigs [16-21]. All these studies used F2 animals as mapping population and only one category of haematological traits (leukocyte traits or erythrocyte traits) were involved. In the present study, we used purebred animals as mapping population and all three categories of haematological traits were analyzed. There is large inconsistency between the QTL for leukocyte and erythrocyte traits reported in the present and the previous studies. For the leukocyte traits, no QTL are in agreement with those reported in the previous studies. In particular, for most traits, the QTL found here and in the previous studies were mapped to different chromosomes. For example, for the trait WBC, we mapped two significant QTL on SSCX, but the QTL for this trait were found on SSC1, SSC8, SSC10, and SSC12 in the previous studies [13-16]. For the erythrocyte traits, only a few QTL are in agreement with those reported in their studies. For HGB and MCHC, we mapped a QTL at 31 cM on SSC2 and a QTL at 19 cM on SSC16, respectively, which overlap those reported by Reiner *et al*. [20]. For RDW, we mapped a QTL at 20 cM on SSC1, which overlaps that reported by Zou *et al*. [21]. Large inconsistency also exists between the results from Reiner *et al*. [20] and those from Zou *et al*. [21]. Both studies involved in mapping QTL for erythrocyte traits in swine and employed the regression approach and a F2 design. 43 and 101 QTL were found by Reiner *et al*. [20] and Zou *et al*. [21], respectively. However, only four are in accordance with each other. A major possible reason for these inconsistencies is that the measurements for the traits are different in different studies. In the study of Edfors-Lilja *et al*. [17], the leukocyte traits were measured immediately before and after the pigs were transported from the farrowing pen to a finishing unit and mixed with non-littermates when they were about three months old. In the study of Zou *et al*. [21], the erythrocyte traits were measured when the pigs were 18, 46, and 240 days old without any stress induction. In the study of Reiner *et al*. [19,20], the leukocyte and erythrocyte traits were measured on the day (when the pigs were approximately 100 days old) of and shortly before infection with 50 000 sporocysts of *Sarcocytis miescheriana of *and on days 14, 28, and 34 after infection. While in the present study, all haematological traits were measured one day before and 14 days after the inoculation with the swine fever vaccine when the pigs were 21 days old. The immune system of pigs is very sensitive to different external stresses. The genetic mechanism of immune responses to different external stress and in different physiological stage is very complex and currently unknown. Therefore, the phenotypes for the same haematological parameters measured in different studies may represent different traits, although they may be highly correlated. Another possible important reason for the discrepancies is that different mapping populations were used in different studies, in which the QTL segregation status may be different. In all cited studies, QTL were detected in crossbred populations while they were detected in purebred populations in the present study.

QTL mapping for platelet traits in swine has not been reported yet. However, QTL for platelet traits had been studied in mice [22,23]. Among the QTL found in the two studies, a QTL for PLT was mapped to 52 Mb [22] and 51 Mb [23] on MMU7, respectively. This region is syntenic with the region on SSC2, where we found a putative QTL (*P *< 0.05, data not shown) for PLT in swine. In addition, Chueng *et al*. [23] mapped a QTL for PLT in mice to 48 Mb on MMU17, which is syntenic with the region of 71 cM on SSC7, where we also found a putative QTL (*P *< 0.05, data not shown) for PLT in swine.

Similar to the results of previous studies [20,21], some QTL located at the same or nearby positions were found to have pleiotropic effects on two to three traits in the present study. These include the QTL located at 38 cM on SSC2 (affecting PDW and PCT), the QTL located at 124 cM on SSC9 (affecting MCH and MCHC), the QTL located at 8 and 9 cM on SSC12 (affecting HGB and HCT), and the QTL located at 51 cM on SSC14 (affecting MCH, MCHC, and RDW). These pleiotropic QTL contribute largely to the correlations between traits. For example, we can imagine a high genetic correlation between traits MCH and MCHC, and indeed, two QTL, one at 124 cM on SSC9 and the other at 51 cM on SSC14, were found having significant effects on both traits.

The candidate genes related to leucocyte traits have been discussed in detail by Wattrang *et al*. [18]. However, none of these genes are located in the QTL regions identified in this study. For the erythrocyte traits, three candidate genes were reported, including the porcine stem cell growth factor receptor (*KIT*) gene [24], the erythropoietin (*EPO*) gene [25], and the erythropoietin receptor (*EPOR*) gene [26]. The *KIT *gene is located at p12-p21on SSC8 and is essential for normal hematopoiesis, melanogenesis, and germ cell development [27]. The *EPO *gene is located at p16-p15 on SSC3 and plays important role in controlling the level of circulating erythrocyte mass by promoting erythroid differentiation and initiating haemoglobin synthesis [28]. The *EPOR *gene is located at q1.2-q2.1 on SSC2 and is critical for erythroid proliferation and differentiation [26]. In the present study, no QTL was found around the position of the *KIT *gene, a putative QTL (*P *< 0.01, data not shown) affecting HGB was mapped between markers SW256 and S0141 on SSC2 which is close to that of the *EPOR *gene, and a QTL affecting MCV was mapped between markers SW2618 and SW836 on SSC3 which is near the position of the *EPO *gene.

## Conclusions

Very few QTL were previously identified for hematological traits of pigs and never in purebred populations. In this study, 35 QTL with FDR < 0.10 were identified for 18 haematological traits: 3 for the leukocyte traits, 28 for the erythrocyte traits, and 4 for the platelet traits. Most of the QTL detected here, in particular the QTL for the platelet traits, have not been reported before. Our results lay important foundation for identifying the causal genes underlying the hematological trait variations in pigs.

## Methods

### Mapping population

The mapping population consisted of 368 purebred piglets of three breeds, including 5 Landrace boar families (15 sows and 87 piglets), 7 Large White boar families (33 sows and 190 piglets), and 4 Songliao Black Pig boar families (15 sows and 90 piglets), respectively. In each litter, 5~7 piglets were randomly selected. Songliao Black Pig is a breed derived from cross of Landrace, Duroc and Ming Pig (a local Chinese pig breed), so it has close genetic link with Landrace. All pigs were raised under standard indoor conditions at the experimental farm of the Institute of Animal Sciences, Chinese Academy of Agricultural Sciences.

### Measurement of phenotypic traits

The piglets were inoculated with the swine fever vaccine on day 21 after birth. Blood samples were collected from each piglet one day before the inoculation (day 20) and two weeks after the inoculation (day 35), respectively. The samples were directly injected into eppendorf tubes containing 60 μl of 20% EDTA in phosphate-buffered saline (PBS). 18 hematologic traits (Table 1) were measured immediately after sample collection using a TEK-II mini automatic hemocyte analysor (Jiangxi Tekang Science and Technology Company, China) with swine specific parameter configuration at Beijing Xiyuan Hospital, China.

### Genetic markers

Genomic DNA was extracted from ear tissues of all piglets and their parents (445 animals in total) using a standard phenol/chloroform method. Microsatellite markers were initially selected from the USDA-MARC porcine reference map http://www.marc.usda.gov/ and were then analyzed for their polymorphisms using DNA from the boars. A final set of 206 informative markers distributed on 19 chromosomes (18 autosomes and chromosome X) was genotyped for all individuals. The total length covered by these markers is 2261.7 centi-Morgans (cM) with an average distance of 11.6 cM between adjacent markers according to the MARC map. The number of markers and their mean polymorphic information content (PIC) on each chromosome are shown in Table 3. For the QTL analysis, the linkage maps of the selected markers were derived directly from the MARC map.

**Table 3:** Number of markers and their mean polymorphic information content (PIC) on each chromosome

Chr.	1	2	3	4	5	6	7	8	9	10	11	12	13	14	15	16	17	18	X
Number of markers	13	9	14	13	12	12	14	12	12	12	7	11	12	11	9	8	8	4	13
Mean PIC	0.63	0.68	0.61	0.54	0.49	0.65	0.63	0.57	0.51	0.53	0.58	0.47	0.55	0.62	0.56	0.55	0.55	0.45	0.51

### QTL analysis

A QTL interval mapping analysis was performed using the variance component approach [29-31] based on a linear mixed model as follows,

where **y **is a vector of the phenotypic values for one of the haematological traits measured on day 35, **a **is a vector of fixed effects including breed (three), sex (two) and sampling season (three), **c **is a vector of the values for that haematological trait measured on day 20, *b *is the regression coefficient, **u **is a vector of polygenic effects, **v **is a vector of QTL allelic effects, **e **is a vector of residuals, **X, Z**, and **T **are incidence matrices for **a**, **u**, and **v**, respectively, **A **is the additive genetic relationship matrix among all individuals,  is the additive polygenetic variance, **Q **is the IBD probability matrix among QTL alleles,  is the QTL allelic variance, **I **is an unit matrix, and  is the residual variance.

The QTL was scanned from left to right at 1 cM intervals along each chromosome. Restricted maximum likelihood (REML) was used to estimate the three variance components in the model and the likelihood ratio (LR) was calculated as test statistic for each particular location on the chromosome

where *L*_MAX _| H_0 _and *L*_MAX _| H_A _are restricted maximum likelihood functions corresponding to the null hypothesis (there is no QTL) and the alternative hypothesis (there is a QTL), respectively.

It is generally regarded that the *LR *follows approximately a χ^2 ^distribution with degrees of freedom between one and two [29]. To be more conservative, we assumed here it follows a χ^2 ^distribution with two degrees of freedom. In addition, in order to avoid the increase of false positive rate caused by multiple tests, the *FDR *(false discovery rate) control approach [32,33] was adopted to determine the significance thresholds based on the *P *values from the χ^2 ^distribution. The positions with *FDR *< 0.05 were considered harboring significant QTL. Let *m *be the total number of tests involved in the analysis and *P*_1 _≤ *P*_2 _≤⋯≤ *P*_*m *_be the ordered observed *P*-values for the *m *tests, the *FDR *for *P*_*i *_is calculated as

Since the QTL analysis was performed at 1 cM intervals along each chromosome, each genome scan is equivalent to 2,261 tests given the assumed genome length. As these tests are not independent, Weller et al [34] recommended as a cautious choice considering the number of markers genotyped. This number was 206 in the present study, leading to the total number of tests *m *= 18 (traits) × 206 (markers) = 3,708.

The LOD-drop off method [34] was used to estimate the confidence intervals of the QTL positions.

Since the variance component method requires normal distribution of the phenotypes, we tested the normality of all traits. It turned out that most of the traits were not normally distributed. The Box-Cox transformation was performed for the traits which severely depart from normal distribution before the QTL analysis was carried out.

## Authors' contributions

YFG and XL are the major executive persons of all jobs of this study, including collection of the phenotypes, marker genotyping, statistical analysis, and drafting this manuscript. ZPW and JZ assisted in the statistical analysis. FH, YRL, SQC, CMQ, SL, XYN and XTQ assisted in phenotype collecting and marker genotyping. QZ planed and supervised the whole study. All authors read and approved the final manuscript.
